# Laparoscopic hiatal hernia repair for treating patients with massive hiatal hernia and iron-deficiency anaemia

**DOI:** 10.1186/s12893-023-02184-3

**Published:** 2023-09-26

**Authors:** Aikebaier Aili, Maimaitiaili Maimaitiming, Yiliang Li, Alimujiang Maisiyiti, Zhi Wang, Yusujiang Tusuntuoheti, Kelimu Abudureyimu

**Affiliations:** 1 Department of Minimally Invasive Surgery, Hernias and Abdominal Wall Surgery, People’s Hospital of Xinjiang Uygur Autonomous Region, Xinjiang Uygur Autonomous Region, Urumqi, China; 2 Xinjiang Clinical Research Center for Gastroesophageal Reflux Disease and Bariatric Metabolic Surgery, Xinjiang Uygur Autonomous Region, Urumqi, China; 3 Research Institute of General and Minimally Invasive Surgery, Xinjiang Uygur Autonomous Region, Xinjiang Uygur Autonomous Region, Urumqi, China; 4 The graduate student institute of Xinjiang Medical University, Urumqi, Xinjiang Uygur Autonomous Region China

**Keywords:** Massive hiatal hernia, Extraoesophageal symptom, Iron-deficiency anaemia, Laparoscopic surgery

## Abstract

**Background:**

Massive hiatal hernias may result in extraoesophageal symptoms, including iron-deficiency anaemia. However, the role played by hiatal hernias in iron-deficiency anaemia is not clearly understood. We examined the prevalence of anaemia in patients with massive hiatal hernias and the frequency of anaemia resolution after laparoscopic hiatal hernia repair at long term follow-up.

**Methods:**

Patients who underwent laparoscopic hiatal hernia repair from June 2008 to June 2019 were enrolled in this study. We collected the patients’ demographic and clinical data from their medical records, and compared the pre-surgical and post-surgical findings (at 1 week and 3 months post-surgery). All patients with adequate documentation underwent post-surgical follow-up to evaluate improvements in clinical symptoms and signs.

**Results:**

A total of 126 patients with massive hiatal hernias underwent laparoscopic hiatal hernia repair. Of these, 35 (27.8%) had iron-deficiency anaemia. Anaemia was resolution in all the patients and they had significantly reduced GERD-Q scores at 3 months postoperatively (*P*<0.01) .The mean follow-up period was 60 months. Iron-deficiency anaemia resolution after hiatal hernia repair was achieved in 93.9% of the patients.

**Conclusion:**

Anaemia is common in patients with massive hiatal hernias, and most of our patients were symptomatic because of their anaemia. Moreover, in patients with massive hiatal hernias, iron-deficiency anaemia resolution is likely after laparoscopic hiatal hernia repair.

## Background

 Massive hiatal hernias (HHs) may result in extraoesophageal symptoms, including iron-deficiency anaemia (IDA) [1]. The association between HHs and anaemia has long been recognised [2]. Some authors define massive HHs as HHs that are greater than 5 cm in size [3]. Although a uniform definition does not exist, a massive(giant) hiatal herniation is defined as any type III, or IV HH [4] and in which more than 30% of the stomach migrates into the chest [5],including short esophageal giant hiatal hernia. Anaemia has been shown to occur more frequently in patients with massive HHs than in those with the more common sliding HHs [6]. The cause of anaemia in patients with HHs has been attributed to linear ulcerations and erosions of the gastric mucosal folds at or near the level of the diaphragm [7].

A Cameron lesion is a source of gastrointestinal bleeding and is typically found during gastroscopy in patients with HH, suggesting that HHs may potentially be a cause of IDA, The highest prevalence of Cameron lesions occur in patients with HHs and the size of the lesion may vary with the size of the HH [8]. The aetiology of the Cameron lesion is thought to involve local mechanical trauma at the herniation site, although focal mucosal ischemia and gastric acid secretion have also been implicated [9]. There appears to be a relationship between HHs and IDA however, the role played by the HH in the pathogenesis of IDA is not clearly understood. This study aimed to assess the prevalence of IDA among patients with massive HH and the frequency of anaemia resolution following laparoscopic HH repair (LHHR).

## Methods

### Study design

Patients with HHs who underwent LHHR at the People’s Hospital of Xinjiang Uygur Autonomous Region (Urumqi, China) between June 2008 to June 2019 were eligible for this study. The patients’ medical records were retrospectively reviewed. The data collected included age, sex, body mass index (BMI), the persistence of anaemia, the presence of oesophageal and gastric ulcers and erosions (as indicated via endoscopy findings), the type of surgical repair, Helicobacter pylori infection status, and the type of fundoplication performed. Anaemia was defined as having a haemoglobin (Hb) level of < 12 g/dl in males, and < 11.0 g/dl in females [10]. We excluded patients who had other identifiable causes of IDA, such as autoimmune diseases, an iron-deficient diet, cancer, and other haematological disorders. To confirm the diagnosis of HH, the patients underwent upper gastrointestinal radiography, gastroscope, and multi-slice spiral computed tomography hernia volume measurement. It is our opinion that evaluation of HHs by oesophageal manometry and pH monitoring is necessary before considering operative interventions, however, a small number of patients either refused this testing or had a reaction, including nausea, rhinallergosis, and cough.

Follow-ups were performed via telephonic interviews or outpatient examinations. The most recent recorded Hb level (at least 3 months after surgery) was used to determine the presence and degree of anaemia. The gastroesophageal reflux disease questionnaire (GERD-Q) [11] score was evaluated. Long term follow-ups were performed to evaluate any improvement in clinical symptoms (including palpitation, pounding in the ears, headaches, and light-headedness) and signs (pallor, glossitis, stomatitis, and angular cheilitis).

The study protocol was approved by the Medical Ethics Committee of the People’s Hospital of Xinjiang Uygur Autonomous Region (NO.2,005,023), and informed consent was obtained from all patients who agreed to participate. All methods were carried out in accordance with relevant guidelines and regulations.

### Surgical procedure

All patients were operated on laparoscopically by two senior surgeons with substantial experience in laparoscopic and gastroesophageal surgery. The operation started with the dissection of the hernia sac from the mediastinum and the reduction of the hernia sac’s contents into the abdomen. After reduction, the complete exposure of the hiatal rim, mobilisation of the oesophagus, and measuring of the diameter of the hiatal hernia, Adequate free hernia sac and oesophagus, and the hiatus was repaired using interrupted sutures made of non-absorbable monofilament suture material. According to the size of the defect and the position of the oesophagus and to prevent oesophageal angulation, the conducted dorsally or with front. If the hiatal closure was assumed to be weak, it was reinforced with mesh. Patients with underwent 360° or 180° or 270° fundoplication.

This study used a self-innovated new type of laparoscopic non-invasive liver retractor (Patent No. ZL201020049682.2), that is suitable for the movement, lifting, and traction of the liver during anti-reflux surgery. Its reasonable structural design, effectiveness in lifting and pulling, and ability to reduce the risk of liver damage during surgery is widely recognized. A separate 2 mm subxiphoid incision was made for insertion of the reverse “7” shaped retractor.

### Statistical analysis

IBM SPSS Statistics for Windows, Version 22.0 (IBM Corp., Armonk, NY, USA) was used for all statistical analyses. The normality for distribution was evaluated via the Shapiro-Wilk test for continuous variables. Continuous variables with a normal distribution were presented as the mean ± standard deviation. Data at baseline and after surgery were tested via paired T tests. The correlations between variables were explored using Spearman’s correlation test. A *P* value of < 0.05 was considered statistically significant.

## Results

A total of 126 patients underwent LHHR at our hospital from June 2008 to June 2019. Of these, 35 (27.8%) were found to be anaemic. Of these anaemic patients, 17 (48.6%) showed symptoms of anaemia or were referred to our hospital specifically for anaemia treatment. Eighteen patients (51.4%) were primarily worked up for gastrointestinal symptoms, including dyspepsia, dysphagia, heartburn, regurgitation, abdominal or chest pain, nausea, and vomiting and were determined to have anaemia based on laboratory findings. Of the patients, 19 (54.3%) had endoscopic evidence of oesophageal and gastric ulcerations and erosions(Cameron lesions), Cameron lesions were not found in 16 (45.7%) patients. The ulcer was treated preoperatively with proton pump inhibitor’s (PPI) and then an oesophageal hiatal hernia repair was performed after the ulcer had resolved. Other baseline patient characteristics are listed in Table 1.

**Table 1:** Patients’ demographic and clinical characteristics (*n* = 35)

Characteristics	Measurements
**Age, years (mean ± SD)**	55.65 ± 11.74
**Sex, n**	
Men	24
Women	11
**BMI, mean ± SD**	28.34 ± 4.17
**Anaemia severity, n**	
Mild	5
Moderate	24
Severe	6
**GERD, n**	
Yes	21
No	14
**Blood transfusion history, n**	
Yes	13
No	22
**Esophagogastric ulceration/erosion, n**	
Yes	19
No	16
***Helicobacter pylori*** **infection, n**	
Positive	11
Negative	24
**Type of fundoplication, n**	
Nissen	27
Toupet	3
Dor	2
None	3

*BMI *Body mass index, *GERD *Gastroesophageal reflux disease, *n *number, *SD *Standard deviation

In this study, all the patients received oral medications and/or blood transfusions as necessary. The mean operation time was 2.78 ± 0.91 h and the mean blood loss during surgery was 32.57 ± 23.56 ml. Additionally, 28 patients underwent mesh reinforcement, and 32 patients underwent fundoplication. The average length of hospital stay was approximately 7 postoperative days.

**Fig. 1:**
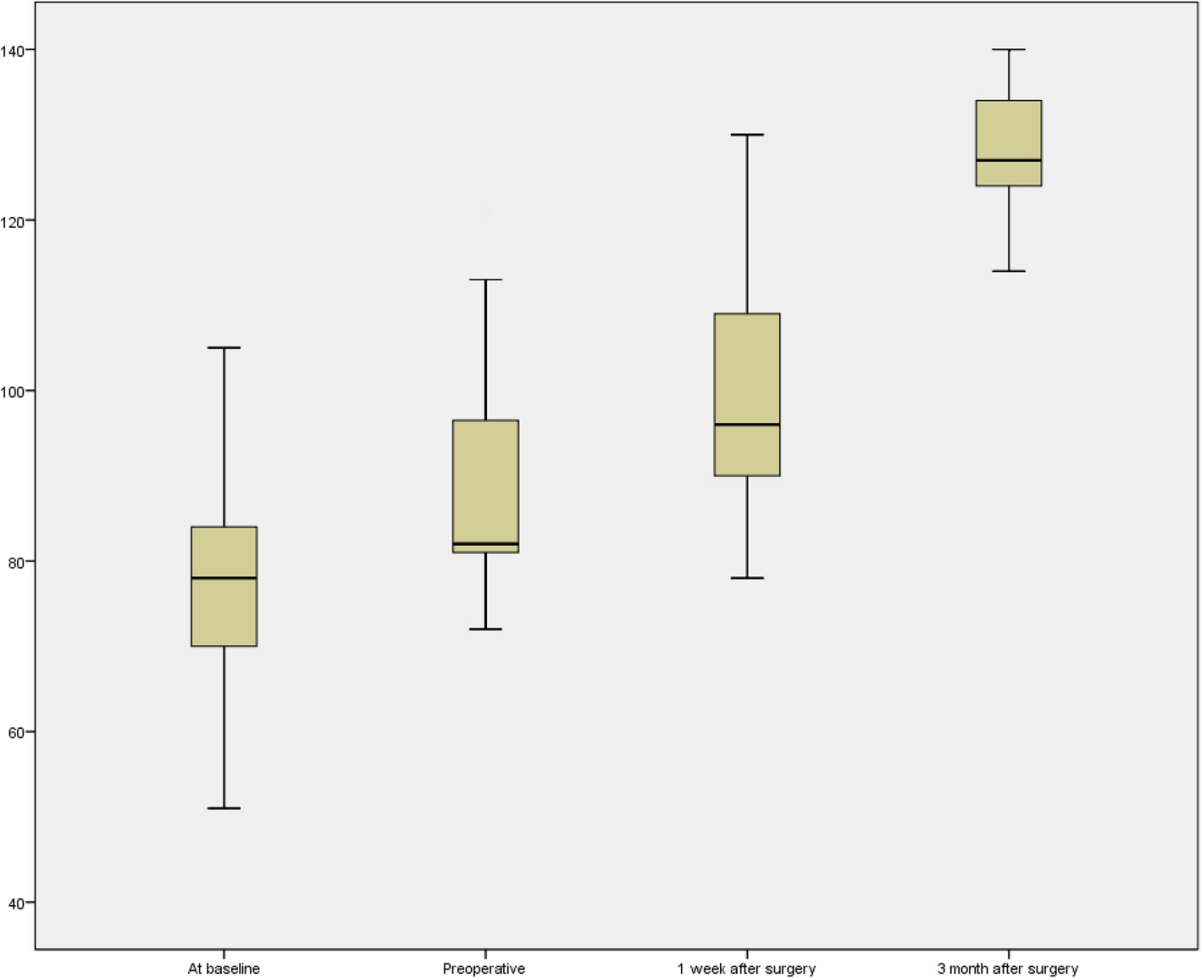
Box and whisker plot depicting the Hemoglobin levels at baseline, Preoperative, 1 week post-operation, and 3 months post-operation

The comparative analysis between the baseline and 1-week postoperative findings showed a significant reduction in the severity of GERD and anaemia after surgery (Table 2) (Fig. 1). The haemoglobin level significantly increased and GERD-Q scores significantly reduced after surgery (Table 3).

**Table 2:** Clinical and biochemical parameters of the study population at 1-week post-surgery

Parameter	At baseline (mean ± SD)	1 week after surgery (mean ± SD)	T value	*P* value
RB(10^12^/L)	3.72 ± 0.719	4.290 ± 0.672	-5.447	<0.001
HB (g/L)	76.57 ± 13.87	99.28 ± 13.86	-11.713	<0.001
PCV	0.295 ± 0.046	0.345 ± 0.046	-6.168	<0.001
MCV (fL)	74.92 ± 9.01	80.92 ± 8.59	-4.865	<0.001
MCH (pg)	22.08 ± 3.99	25.29 ± 3.85	-5.335	<0.001
MCHC (g/L)	287.94 ± 23.71	299.66 ± 28.14	-2.184	0.036

*Hb *Haemoglobin, *PCV *Packed cell volume, *MCV *Mean corpuscular volume, *MCH *Mean corpuscular haemoglobin, *MCHC *Mean corpuscular haemoglobin concentration, *RB *Red blood cell, *SD *Standard deviation

**Table 3:** Clinical and biochemical parameters of the study population at 3 months postoperatively

Variables	At baseline (mean ± SD)	3 months after surgery (mean ± SD)	T value	*P* value
GERD-Q score	12.11 ± 1.32	6.37 ± 1.08	18.830	<0.001
HB (g/L)	76.57 ± 13.87	128.28 ± 6.92	-21.744	<0.001

*GERD-Q *Gastroesophageal reflux disease questionnaire

All patients with anaemia had the adequate amount of documentation required to undergo follow-ups via telephonic interviews. The mean follow-up period was 60 months (range, 28–121 months).

During the study period, two patients were lost to follow-up. Thirty-three patients were given a long-term follow-up, and of them the remaining 31 patients’ anaemia resolved postoperatively (no clinical symptoms and signs and the patient did not receive any oral anaemia medication and/or blood transfusion postoperatively). The frequency of resolution of anaemia was 93.9%(31/33), and the remaining 2 patients’ had a recurrence of the HH, and required oral iron supplementation. One of these patients was a 74-year-old male whose baseline Hb was 72 g/L and it increased to 114 g/L at 3 months postoperatively. After 6 years follow-up, this patient’s Hb was 98 g/L. In this patient, the stomach could not be completely returned to the abdominal cavity during the operation and the gastric wall and diaphragm were sutured and fixed without mesh. The other patient was a 78-year-old male and at baseline his Hb was 56 g/L, 3 months postoperatively his Hb was 124 g/L, and his Hb was 81 g/L at 5 year follow-up.In this patient, fixed without mesh, and he was complicated with chronic obstructive pulmonary disease, Liver cirrhosis, Personal history of bladder cancer.

## Discussion

Anaemia is a more common complication in patients with HHs and the prevalence of anaemia among patients with HHs is as high as 33% [12]. A linear gastric erosion, or Cameron lesion, is often associated with large diaphragmatic hernias and chronic blood loss anaemia [7]. In our study, the 35 (27.8%) patients with massive HHs were confirmed to have IDA, and of the anaemic patients, 19 (54.3%) had endoscopic evidence of oesophageal and gastric ulcerations and erosions. This may indicate that the anaemia was caused by these Cameron lesions near the level of the diaphragm. Interestingly, Cameron lesions were not found in 16 (45.7%) patients. Iron-deficiency anaemia occurs more frequently in those with large hernias (> 5 cm) than in those with small hernias (< 3 cm) [13]. We believe that this may be due to the pressure difference between the thoracic and abdominal cavities. In large hernia cases, the stomach gets sucked into the bottom portion of the thoracic cavity and this portion of the stomach is unable to properly participate in digestion, thereby seriously affecting the secretion of pepsin and gastric acid. This leads to malnutrition and iron absorption disorders, which, in turn, eventually results in chronic IDA.

Previous studies have demonstrated the near-complete resolution of anaemia via hernia repair. Haurani et al. [14] studied 66 patients with anaemia who also had a paraesophageal hernia and underwent surgical repair; the vast majority (85%) of those with ulceration or erosion had resolution of their anaemia postoperatively, indicative of apparent healing. In this study, it was not PPI given to all patients post operatively, and patients with severe oesophageal and gastric ulcerations and erosions took PPI drugs before surgery and underwent surgery after the ulcer improved or cured.And all the patients received oral medications and/or blood transfusions as necessary and consequently the early postoperative Hb improvements may be due to the oral medications or/and transfusions rather than the surgery. However, our long term follow-up results show a similar rate (93.9%) of anaemia resolution after surgical repair, which can be attributed to the fact that, following the operation, the lesions are healed, the stomach is returned to the normal position, and the pressure difference between the thorax and abdomen is eliminated.

Further, the proton pump inhibitor drugs were much more effective for those whose symptoms were less severe or whose HH was small. In a patient for whom the effect of medication is inadequate or the patient requires long-term medication, surgical treatment should be considered, especially for large hernias and patients with signs of reflux-induced thinning and bleeding of the gastroesophageal lining [15]. Proton pump inhibitor drugs, with or without iron supplementation, are recommended for the treatment of patients who are anaemic due to massive hernias [16] however, the indications for this treatment are not permanent, the addition of surgery normalizes Hgb, appears durable, allows cessation of medication, and improves quality of life [1]. Therefore, the patients who are anaemic due to massive hernias should undergo surgical repair, even if they are asymptomatic.

Most patients with massive HHs also have accompanying gastroesophageal and respiratory symptoms, further supporting the need to correct their defects through surgery. Experienced surgeons [17, 18] have proven that LHHR is a safe and effective approach. Our results support the use of surgical repair for treating massive HHs in patients with symptomatic anaemia, especially for those with oesophageal and gastric ulcerations and erosions, since most of our patients achieved anaemia resolution. Additionally, we used the GERD-Q to assess patients’ symptoms after undergoing laparoscopic anti-reflux surgery and our results show that LHHR can improve patients’ satisfaction with their quality of life, as the majority were able to discontinue anti-reflux therapy and iron supplementation after surgery. This discontinuation offers financial benefits to both patients and the healthcare system and reduces the incidence of unnecessary pharmacologic therapy and adverse side effects linked to the long-term use of anti-reflux medication and iron supplementation.

Our study has several limitations, including the retrospective nature of our data, our small study population, and that this study was conducted at a single-centre may be led to selective bias. Our long term follow-up was not standardized and did not provide complete results.

## Conclusions

Anaemia is a common finding in patients with massive HHs. Most of these patients were symptomatic because of their anaemia, and such patients are likely to present with endoscopic evidence of oesophageal and gastric ulcerations and erosions. Furthermore, anaemic patients who have evidence of Cameron lesions are likely to show symptoms of anaemia, and these patients, especially, are likely to achieve anaemia resolution after undergoing LHHR. In the absence of other identifiable causes of reduced Hb levels, massive HHs should be considered as the cause of the anaemia, and patients should be further evaluated to confirm the presence of hernias. Such patients are likely to benefit from the anaemia resolution and quality of life improvements provided by the surgical repair of their hernia.

## Data Availability

The datasets used and/or analyzed during the current study are available from the corresponding author on reasonable request.

## Abbreviations

BMI: Body mass index
GERD-Q: Gastroesophageal reflux disease questionnaire
Hb: Haemoglobin
HH: Hiatal hernia
IDA: Iron-deficiency anaemia
LHHR: Laparoscopic hiatal hernia repair
PPI: Proton pump inhibitor

## References

[1] Laliberte AS, Brandabur JJ, Chang SC et al. Changes in hemoglobin levels in patients with hiatal hernia and Anemia demonstrates a durable resolution when surgery Utilized[J]. 2021.10.1177/26345161211025277.

[2] Ruhl CE, Everhart JE. Relationship of iron-deficiency anemia with esophagitis and hiatal hernia: hospital findings from a prospective, population-based study[J]. 2001, 96(2):322–610.1016/s0002-9270(00)02306-6. 10.1111/j.1572-0241.2001.03513.x 11232670

[3] Oelschlager BK, Pellegrini CA, Hunter J, Soper N, Brunt M, Sheppard B (2006). Biologic prosthesis reduces recurrence after laparoscopic paraesophageal hernia repair: a multicenter, prospective, randomized trial. Ann Surg.

[4] Duranceau A (2016). Massive hiatal hernia: a review. Dis Esophagus.

[5] Mitiek MO, Andrade RS (2010). Giant hiatal Hernia[J]. Ann Thorac Surg.

[6] Windsor CW, Collis JL (1967). Anaemia and hiatus hernia: experience in 450 patients. Thorax.

[7] Cameron AJ, Higgins JA (1986). Linear gastric erosion. A lesion associated with large diaphragmatic hernia and chronic blood loss anemia. Gastroenterology.

[8] Gray DM, Kushnir V, Kalra G, Rosenstock A, Alsakka MA, Patel A (2015). Cameron lesions in patients with hiatal hernias: prevalence, presentation, and treatment outcome. Dis Esophagus.

[9] Panzuto F, Di Giulio E, Capurso G, Baccini F, D’Ambra G, Delle Fave G (2004). Large hiatal hernia in patients with iron deficiency anaemia: a prospective study on prevalence and treatment. Aliment Pharmacol Ther.

[10] Guo J, Zheng C, Xiao Q, Gong S, Zhao Q, Wang L (2015). Impact of anaemia on lung function and exercise capacity in patients with stable severe chronic obstructive pulmonary disease. BMJ Open.

[11] Gong Y, Zeng Q, Yan Y, Han C, Zheng Y (2019). Association between lifestyle and gastroesophageal reflux disease questionnaire scores: a cross-sectional study of 37 442 chinese adults. Gastroenterol Res Pract.

[12] Patel HJ, Tan BB, Yee J, Orringer MB, Iannettoni MD (2004). A 25-year experience with open primary transthoracic repair of paraesophageal hiatal hernia. J Thorac Cardiovasc Surg.

[13] Bernardo RJ, Portocarrero JP, Tagle M (2012). [Cameron lesions: clinical experience]. Revista de Gastroenterologia del Peru: Organo Oficial de la Sociedad de Gastroenterologia del Peru.

[14] Haurani C, Carlin AM, Hammoud ZT, Velanovich V (2012). Prevalence and resolution of anemia with paraesophageal hernia repair. J Gastrointest Surg.

[15] Yu HX, Han CS, Xue JR, Han ZF, Xin H (2018). Esophageal hiatal hernia: risk, diagnosis and management. Expert Rev Gastroenterol Hepatol.

[16] Moskovitz M, Fadden R, Min T, Jansma D, Gavaler J (1992). Large hiatal hernias, anemia, and linear gastric erosion: studies of etiology and medical therapy. Am J Gastroenterol..

[17] Pierre AF, Luketich JD, Fernando HC, Christie NA, Schauer PR. Results of laparoscopic repair of giant paraesophageal hernias: 200 consecutive patients[J]. The Annals of Thoracic Surgery. 2003;74:1909-15; discussion 1915–1916. 10.1016/S0003-4975(02)04088-2. 10.1016/s0003-4975(02)04088-2 12643372

[18] Andujar JJ, Papasavas PK, Birdas T, Robke J, Raftopoulos Y, Gagné DJ, Caushaj PF (2004). Laparoscopic repair of large paraesophageal hernia is associated with a low incidence of recurrence and reoperation. Surg Endosc.

